# Impact of hereditary angioedema attacks on health-related quality of life and work productivity^☆^

**DOI:** 10.1016/j.waojou.2025.101083

**Published:** 2025-07-28

**Authors:** Maeve O'Connor, Paula J. Busse, Timothy J. Craig, Cristine Radojicic, H. James Wedner, Sherry Danese, Julie Ulloa, Vibha Desai, Tomas Andriotti, Paul K. Audhya, Sandra Christiansen

**Affiliations:** a Integrative Immunology Care, LLC, Charlotte, NC, USA; b Allergy, Asthma, & Immunology Research Institute, Charlotte, NC, USA; c Consortium of Independent Immunology Clinics, Dallas, TX, USA; d Mount Sinai School of Medicine, New York, NY, USA; e Penn State University, Hershey, PA, USA; f Vinmec International Hospital, Times City, Hanoi, Vietnam; g Duke Health, Durham, NC, USA; h Washington University School of Medicine, St. Louis, MO, USA; i Outcomes Insights, Inc., Agoura Hills, CA, USA; j Providence Internal Medicine, Spokane, WA, USA; k KalVista Pharmaceuticals, Inc., Boston, MA, USA; l University of California San Diego, La Jolla, CA, USA

**Keywords:** Hereditary angioedema, Long-term prophylaxis, On-demand therapy, Work productivity, Quality of life

## Abstract

**Background:**

Hereditary angioedema (HAE) negatively impacts health-related quality of life (HRQoL). Few studies have characterized impairments to HRQoL, work productivity, and well-being during and following HAE attacks.

**Methods:**

Patients with ≥1 HAE attack in the previous 3 months were recruited by the United States (US) HAE Association (HAEA) to complete an online survey. Respondents were categorized into those who did (Treated Cohort) and did not (Untreated Cohort) manage their last attack with on-demand treatment (OD). Adapted versions of the EuroQol–Five Dimensions–Five Levels (EQ-5D-5L), EuroQol Visual Analogue Scale (EQ VAS), and Work Productivity and Activity Impairment–General Health assessments were used.

**Results:**

Attacks negatively impacted HRQoL and social and physical aspects of well-being in the Treated (N = 94) and Untreated (N = 20) Cohorts. In the Treated Cohort, mean EQ-5D-5L index score was 0.849 “Today” and 0.568 “During the last treated attack,” with the latter score indicating substantial impairment in HRQoL. Mean EQ-5D-5L index scores trended lower as time to OD increased, with mean scores of 0.667 for those treating <1 h and 0.593-0.504 for those treating ≥1 to <8 h from attack onset. The percentage of patients with mild attacks decreased as time to OD increased (from 50% for those treating <1 h to 17–33% for those treating ≥1 h from attack onset), and mean EQ-5D-5L index scores decreased as attack severity increased (mild, 0.742; severe, 0.444). Generally, mean EQ VAS scores decreased as time to OD increased, from 60.1 in those treating <1 h to 53.3 in those treating ≥8 h from attack onset. In the week following attack onset, average levels of absenteeism, presenteeism, and overall work impairment were 15%, 35%, and 39%, respectively. Despite attacks being of milder severity, declines in HRQoL (mean EQ-5D-5L and EQ VAS scores “During the last attack”: 0.661 and 73.0, respectively) and impairments in work productivity (mean level of absenteeism, presenteeism, and overall work impairment: 12%, 32%, and 36%, respectively) were also observed in the Untreated Cohort.

**Conclusions:**

Late-treated and untreated attacks were associated with reduced HRQoL and work productivity, driven by attack severity. The negative impact of attacks may be reduced by increasing compliance with HAE guidelines (ie, consider OD for all attacks and treat as soon as possible), and by addressing barriers to OD in general and early treatment in particular.

## Introduction

Hereditary angioedema (HAE) is a rare genetic disease affecting an estimated 1 in 50,000–90,000 persons globally^1^, ^2^, ^3^ and ∼6000 people in the United States (US).^4^ HAE is associated with unpredictable, painful, and debilitating attacks of tissue (subcutaneous and submucosal) swelling that can be life-threatening if affecting the larynx.^5^ HAE in general^6^, ^7^, ^8^, ^9^, ^10^ and attacks in particular^11^ are known to negatively impact health-related quality of life (HRQoL).

To mitigate the adverse effects of attacks, HAE guidelines recommend that patients (1) consider using on-demand treatment (OD) to manage all attacks, irrespective of location or severity, (2) treat attacks as early as possible after onset to halt progression and reduce attack severity and duration, and (3) always carry sufficient OD to manage ≥2 attacks, as triggers are often unknown.^4^^,^^12^^,^^13^ All currently approved ODs are administered via either intravenous (IV) infusion or subcutaneous (SC) injection. Patients with HAE may experience emotional (eg, stress, anxiety, needle fears), logistical (eg, transport/portability of treatment, venous access, dexterity to self-treat at the time of the attack), and/or clinical (eg, injection site reactions) issues with the self-injection of parenterally administered OD.^14^, ^15^, ^16^, ^17^ As such, it is not surprising that not all patients treat attacks in a timely manner and that some attacks are not treated at all.^14^^,^^18^^,^^19^

Although several international studies have demonstrated the negative impact that HAE has on patient HRQoL,^6^, ^7^, ^8^, ^9^, ^10^ only a limited number have characterized HRQoL both during and following attacks,^20^^,^^21^ and none has assessed the effect of OD on patient well-being. As such, the objective of the present analysis was to evaluate HRQoL, work productivity, and activity impairment during and following attacks, both treated and untreated, in US patients with HAE, including those using long-term prophylaxis (LTP); the impact of the time to OD from attack onset on patient-reported outcomes (PROs) was also explored. Since administration route is known to influence the choice and timing of OD,^14^^,^^22^^,^^23^ we also examined outcomes in patients who treated their last attack with an SC versus IV formulation of OD.

## Methods

### Study design

Patients aged ≥12 years with physician-diagnosed HAE-C1 esterase inhibitor (C1INH)-Type1 or HAE-C1INH-Type2 who experienced ≥1 attack in the previous 3 months and had access to OD were recruited between April 2023 and June 2023 by the US HAE Association (HAEA) to complete a 20-min online survey.^19^ The survey comprised 74 questions related to demographics; comorbidities; attack frequency and location in the past 12 months; last attack characteristics and experiences; treatment use (if any) at the time of the last attack; and HRQoL, work productivity, and activity impairment during and following the last attack.

### Study cohorts

Respondents were categorized into 2 cohorts, those who treated their last attack with OD (Treated Cohort) and those who did not treat their last attack with OD (Untreated Cohort). To describe the impact of treatment delays, outcomes were measured in subgroups defined by the time between attack onset and OD administration (<1 h, 1 to < 2 h, 2 to < 5 h, 5 to < 8 h, and ≥8 h). In unadjusted analyses, attack severity (as reported by the patient) at the time of OD was examined in subgroups defined by the amount of time that elapsed between attack onset and treatment. HRQoL was evaluated in separate unadjusted analyses in subgroups defined by attack severity at the time of OD. For each cohort, outcomes were described in those who had been prescribed OD only and those who had been prescribed non-androgen LTP in addition to OD (LTP + OD) at the time of their last attack. The Treated Cohort included respondents aged ≥12 years, whereas the Untreated Cohort was restricted to respondents aged ≥18 years. Additional analyses based on age (adolescents or adults) and route of OD administration (IV or SC) were performed on the Treated Cohort.

### Survey and outcome measures

#### Adapted EuroQol–Five Dimensions–Five Levels (EQ-5D-5L) and EuroQol Visual Analogue Scale (EQ VAS)

The EQ-5D-5L was used to assess physical and mental HRQoL "Today" (ie, current HRQoL) and was adapted to include recall of HRQoL “During the last treated/untreated attack.“^24^ The EQ-5D-5L index score, a measure of overall health state, is generated based on scores in 5 different HRQoL domains: mobility, self-care, usual activities, pain/discomfort, and anxiety/depression. EQ-5D-5L scores range from −0.573 (Lowest possible health state) to 1 (Best possible health state).^25^ The EQ VAS was used to measure overall health status "Today" and “During the last treated/untreated attack,” with scores ranging from 0 (Worst imaginable health state) to 100 (Best imaginable health state).^24^ The mean US norm for online respondents to the EQ-5D-5L and EQ VAS is 0.800 and 74.6, respectively.^26^ A difference of 0.078 on the EQ-5D-5L is considered clinically important for the United States.^27^

#### Adapted HAEA-QoL questionnaire version 2 (HAEA-QoLv2)

The HAEA-QoLv2 was used to assess physical and social HRQoL. Unlike the EQ-5D-5L and EQ VAS, the HAEA-QoLv2 was designed specifically for use in patients with HAE. Questions from the HAEA-QoLv2 were adapted from asking patients to qualify the HRQoL effects of HAE in general to asking them to focus on the HRQoL effects of the last attack. The social impact of HAE (feeling embarrassed, socially isolated, etc.) was measured via a 5-point Likert scale, with scores ranging from 1 (Strongly disagree) to 5 (Strongly agree). The physical impact of HAE on energy level, sleep, and activity level was measured on a scale of 1 (Not at all) to 4 (A lot/severe).^28^

#### Adapted work productivity and activity Impairment–General Health (WPAI-GH)

The WPAI-GH, a six-item instrument, was used to measure impairments in work and activities due to an HAE attack. The time frame for the questionnaire was adapted such that respondents were asked to rate work productivity and activity impairment “During the 7 days that followed the start of the last treated/untreated HAE attack*”* rather than “During the past 7 days.”

All adapted HRQoL, work productivity, and activity impairment measures were described by attack severity to assess validity.

### Statistics

The target sample size for the Treated Cohort was 100 patients, with a minimum of 50 receiving OD only (and maximum of 50 receiving LTP + OD) at the time of the last treated attack, 20 adolescents (12–17 years) and 80 adults (≥18 years). Descriptive analyses were performed, with outcomes summarized as numbers, percentages, means, and standard deviations (SDs), as appropriate, for the Treated and Untreated Cohorts and for each subgroup.

## Results

### Respondents

A total of 94 respondents were included in the Treated Cohort, and 20 were included in the Untreated Cohort. Demographics, clinical characteristics, and last treatment attack details have been described.^19^

Briefly, 91% (86/94) of respondents in the Treated Cohort experienced their most recent attack in the previous 30 days, with 29% (27/94) describing the severity of their attack at the time of OD as “Mild,” 55% (52/94) as “Moderate,” and 16% (15/94) as “Severe” or “Very severe.“^19^ In total, 46% (43/94) of respondents in the Treated Cohort had been prescribed OD only, and 54% (51/94) had been prescribed LTP + OD at the time of their last attack. In the Treated Cohort, the majority of respondents were adults (85% [80/94]) and most (65% [61/94]) were prescribed an SC formulation of OD. The mean (SD) time to treatment was 2.9 (3.5) hours in the SC subgroup and 5.8 (9.6) hours in the IV subgroup.

In the Untreated Cohort, 75% (15/20) of respondents experienced their most recent attack within the past 30 days, with 70% (14/20) describing their attack at onset as “Mild,” 25% (5/20) as “Moderate,” and 5% (1/20) as “Severe.“^19^ A total of 45% (9/20) of respondents in the Untreated Cohort had been prescribed OD only, and 55% (11/20) had been prescribed LTP + OD at the time of their last attack. All respondents in the Untreated Cohort were adults.

### Treated cohort

#### EQ-5D-5L index scores

**Fig. 1:**
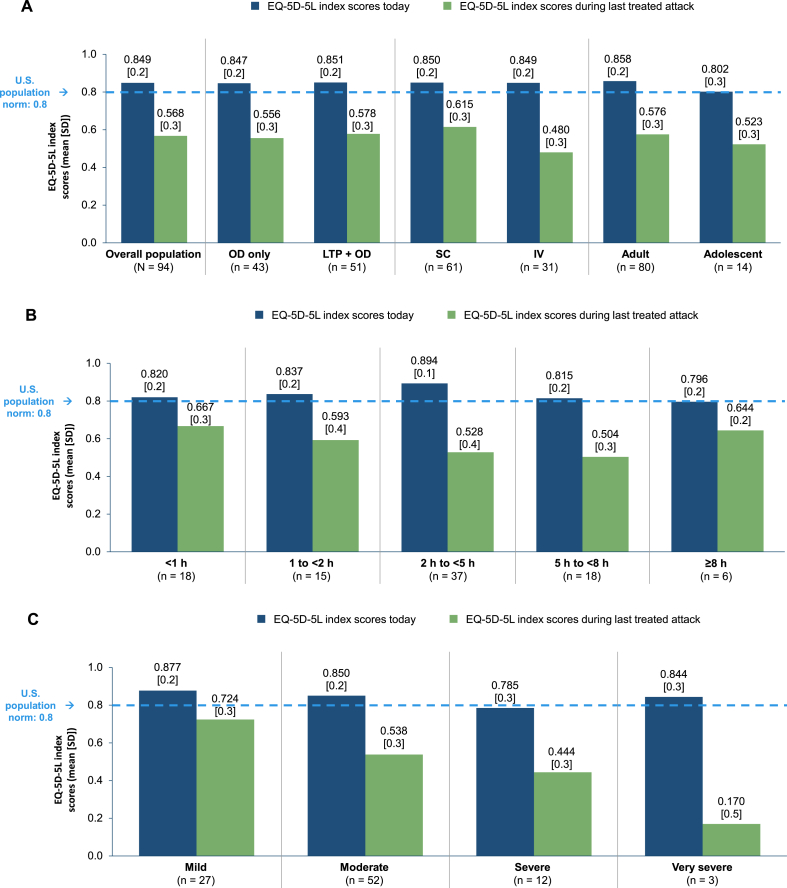
EQ-5D-5L index scores in the Treated Cohort and in subgroups defined by prescribed treatment, administration mode, and age (A); by time to OD (B); and by attack severity at the time of OD (C). EQ-5D-5L, EuroQol-Five Dimensions-Five Levels; LTP, long-term prophylaxis; OD, on-demand treatment; SD, standard deviation.

In the Treated Cohort, the mean (SD) EQ-5D-5L index score was 0.849 (0.2) “Today” and 0.568 (0.3) “During the last treated attack” (Fig. 1A), with the latter score indicative of substantial impairment in HRQoL. Mean EQ-5D-5L index scores generally decreased as the time to administration of OD increased. The mean (SD) index score was 0.667 (0.3) in patients who treated <1 h of attack onset and 0.504 (0.3) in those who treated 5 to < 8 h of attack onset (Fig. 1B). As the severity of the last treated attack increased, so did the negative impact on health status: the mean (SD) EQ-5D-5L index score was 0.724 (0.3) in the “Mild” subgroup, 0.538 (0.3) in the “Moderate” subgroup, 0.444 (0.3) in the “Severe” subgroup, and 0.170 (0.5) in the “Very severe” subgroup (Fig. 1C). The mean EQ-5D-5L index score “Today” was 0.847 in the OD-only subgroup, 0.851 in the LTP + OD subgroup, 0.858 in adults, and 0.802 in adolescents. For these subgroups, the mean EQ-5D-5L index scores “During the last treated attack” were 0.556, 0.578, 0.576, and 0.523, respectively (Fig. 1A). Among patients who administered an SC formulation of OD, the mean (SD) EQ-5D-5L index scores “Today” and “During the last treated attack” were 0.850 (0.2) and 0.615 (0.3), respectively. The corresponding values in patients who administered an IV formulation of OD were 0.849 (0.2) and 0.480 (0.3).

#### EQ VAS score

**Fig. 2:**
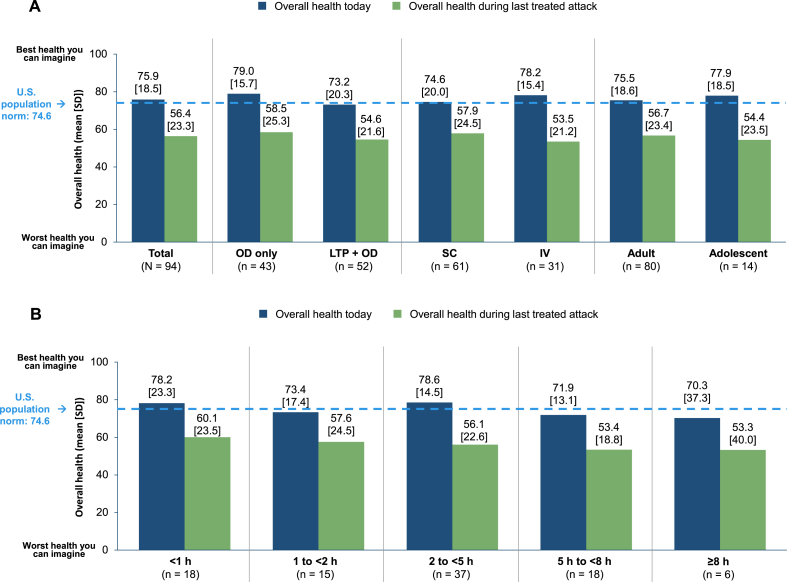
EQ VAS score in the Treated Cohort and in subgroups defined by prescribed treatment, administration mode, and age (A) and by the time to OD (B). EQ VAS, EuroQol Visual Analogue Scale; LTP, long-term prophylaxis; OD, on-demand treatment; SD, standard deviation; VAS, visual analog scale.

In the Treated Cohort, the mean (SD) overall health score “During the last treated attack” was nearly 20 points lower than the score “Today” (56.4 [23.3] and 75.9 [18.5], respectively) (Fig. 2A). The mean EQ VAS score at the time of the last treated attack decreased as the time to OD increased. The mean (SD) EQ VAS score was 60.1 (23.5) in patients who treated within <1 h of attack onset, 53.4 (18.8) in those who treated within 5 to < 8 h of attack onset, and 53.3 (40.0) in those who treated ≥8 h of attack onset (Fig. 2B).

The mean (SD) EQ VAS score “Today” was 79.0 (15.7) in the OD-only subgroup, 73.2 (20.3) in the LTP + OD subgroup, 75.5 (18.6) in adults, 77.9 (18.6) in adolescents, 74.6 (20.0) in the SC subgroup, and 78.2 (15.4) in the IV subgroup (Fig. 2A). In comparison with “Today,” mean (SD) scores “During the last treated attack” were approximately 20 points lower for the OD-only subgroup (58.5 [25.3]), the LTP + OD subgroup (54.6 [21.6]), adults (56.7 [23.4]), adolescents (54.4 [23.5]), the SC subgroup (57.9 [24.5]), and the IV subgroup (53.5 [21.2]).

#### HAEA-QoLv2 outcomes

**Fig. 3:**
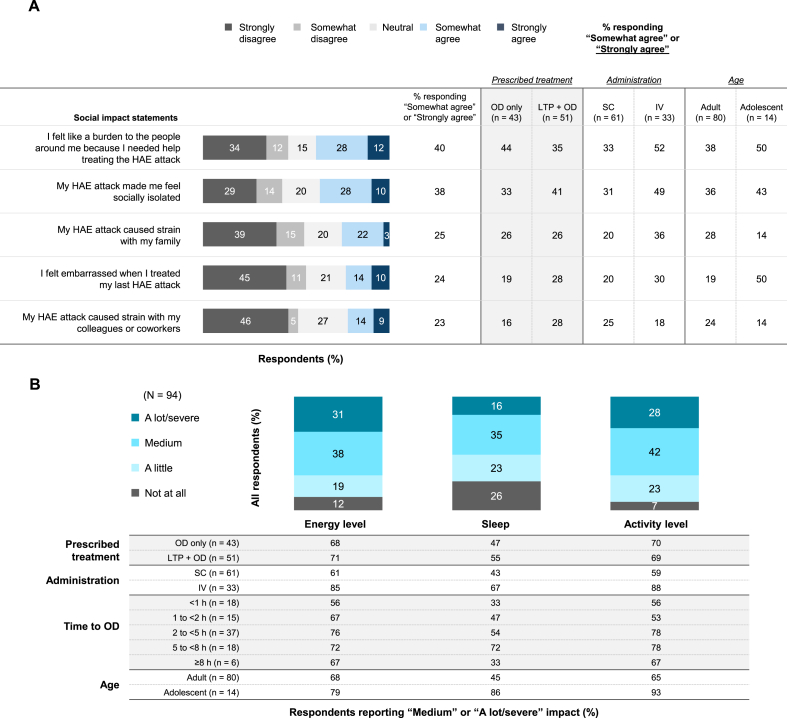
HAEA-QoLv2 outcomes for social impact (A) and physical impact (B) in the Treated Cohort. Note: Values < 5% are not labeled. HAE, hereditary angioedema; HAEA-QoLv2, Hereditary Angioedema Association-Quality of Life Questionnaire version 2; OD, on-demand treatment; LTP, long-term prophylaxis.

In total, 40% (37/94) of respondents in the Treated Cohort “Somewhat agreed” or “Strongly agreed” with the statement “I felt like a burden to the people around me because I needed help treating the HAE attack,” and 38% (35/94) “Somewhat agreed” or “Strongly agreed” with the statement “My HAE attack made me feel socially isolated” (Fig. 3A). In terms of physical HRQoL, approximately 70% of respondents felt that their last HAE attack had a “Medium” or “A lot/severe” negative impact on both their energy level and activity level (Fig. 3B). Just over half of respondents (51% [48/94]) reported that their last HAE attack had a “Medium” (35% [33/94]) or “A lot/severe” (16% [15/94]) impact on their sleep. In general, there was a trend for treatment delays to have a greater negative impact on energy level, sleep, and activity level. This trend did not hold for the subgroup of patients treated ≥8 h from attack onset, likely because of the small number of such respondents (n = 6). Respondents with attacks of greater severity at treatment experienced greater negative impacts on all 3 physical parameters (energy level, sleep, and activity level) of the HAEA-QoLv2 **(**Supplementary Fig. S1**)**.

As to the social impact of attacks in key subgroups, 44% (19/43) of respondents prescribed OD only, 35% (18/51) of those prescribed LTP + OD, 38% (30/80) of adults, 50% (7/14) of adolescents, 33% (20/61) of those in the SC subgroup, and 52% (17/33) of those in the IV subgroup “Somewhat agreed” or “Strongly agreed” with feeling like a burden to others (Fig. 3A). A total of 33% (14/43), 41% (21/51), 36% (29/80), 43% (6/14), 31% (19/61), and 48% (16/33), respectively, “Somewhat agreed” or “Strongly agreed” with feeling socially isolated because of their attack. Additionally, 50% (7/14) of adolescents “Somewhat agreed” or “Strongly agreed” that they felt embarrassed when treating their last attack. In the OD-only subgroup, 68% (29/43), 47% (20/43), and 70% (30/43) of respondents reported that their last treated attack had a “Medium” or “A lot/severe” negative impact on their energy level, sleep, and activity level, respectively (Fig. 3B). The corresponding values in the LTP + OD subgroup were 71% (36/51), 55% (28/51), and 69% (35/51). The majority of adolescents reported “Medium” or “A lot/severe” negative impact on their energy level, sleep, and activity level (79% [11/14], 86% [12/14), and 93% [13/14], respectively). The corresponding values were 61% (37/61), 43% (26/61), and 59% (36/61) in the SC subgroup and 85% (28/33), 67% (22/33), and 88% (29/33) in the IV subgroup.

#### Adapted WPAI-GH

**Fig. 4:**
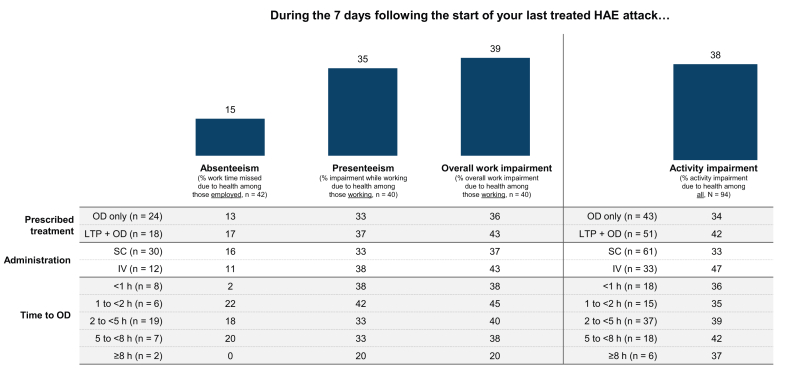
Adapted WPAI-GH in the Treated Cohort. HAE, hereditary angioedema; LTP, long-term prophylaxis; OD, on-demand treatment; WPAI-GH, Work Productivity and Activity Impairment-General Health.

In the Treated Cohort, the mean value for attack-related activity impairment was 38% (Fig. 4). The impact of attacks on work productivity was limited to the 42 adults who reported being employed (full- or part-time) at the time of their last treated attack. Characteristics of employed respondents were generally comparable with those in the overall Treated Cohort, but employed respondents were more likely to have private or commercial insurance (86% [36/42] vs 69% [65/94]) and be prescribed OD only (57% [24/42] vs 46% [43/94]) **(**Supplementary Table S1**)**. At the time of OD, 62% (26/42) of employed respondents described their attack as “Moderate.” Respondents with private or commercial insurance were more likely than those with Medicare/Medicaid to delay treatment because they wanted to save their OD for a severe attack (34% vs 14%, respectively).

The last treated attack had a measurable effect on work productivity (Fig. 4). In the 7 days following attack onset, the average rates of absenteeism (work missed due to HAE), presenteeism (impairment while working due to HAE), and overall work impairment were 15%, 35%, and 39%, respectively. In patients who treated <1 h of attack onset, the average rates of absenteeism, presenteeism, and overall work impairment were 2%, 38%, and 38%, respectively; the corresponding values in patients who treated 5 to <8 h of attack onset were 20%, 33%, and 38%. In the OD-only subgroup, the average rates of absenteeism, presenteeism, and overall work impairment were 13%, 33%, and 36%, respectively; the corresponding values in the LTP + OD subgroup were 17%, 37%, and 43%. The average rates of absenteeism, presenteeism, and overall work impairment were 16%, 33%, and 37%, respectively, in the SC subgroup and 11%, 38%, and 43%, respectively, in the IV subgroup. Greater attack severity at the time of OD was associated with reduced work productivity. For respondents with “Mild,” “Moderate,” and “Severe” attacks, the mean level of absenteeism was 5%, 16%, and 35%; the mean level of presenteeism was 29%, 32%, and 68%; and mean overall work impairment was 32%, 36%, and 75%, respectively.

### Untreated cohort

#### EQ-5D-5L index scores

**Fig. 5:**
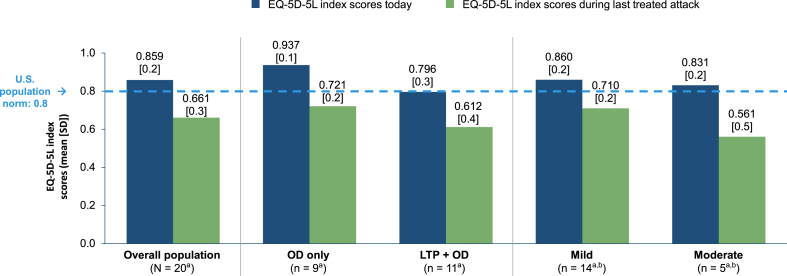
EQ-5D-5L index scores in the Untreated Cohort and in subgroups defined by prescribed treatment and by attack severity at onset. ^a^Because of the small sample size, data should be interpreted with caution. ^b^1 patient with a severe attack was not reported. EQ-5D-5L, EuroQol-Five Dimensions-Five Levels; LTP, long-term prophylaxis; OD, on-demand treatment; SD, standard deviation.

In the Untreated Cohort, the mean (SD) EQ-5D-5L index score was 0.859 (0.2) “Today” and 0.661 (0.3) “During the last untreated attack” (Fig. 5). Respondents who categorized the severity of their attack as “Moderate” at onset reported a lower mean (SD) EQ-5D-5L index score “During the last untreated attack” than did those with a “Mild” attack at onset (0.561 [0.5] vs 0.710 [0.2]). The mean (SD) EQ-5D-5L index score “During the last untreated attack” was 0.721 (0.2) in the OD-only subgroup and 0.612 (0.4) in the LTP + OD subgroup.

#### EQ VAS score

**Fig. 6:**
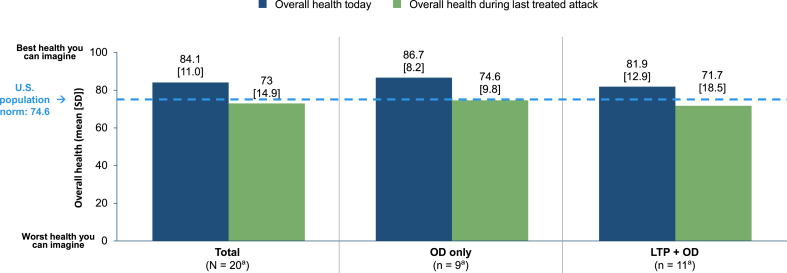
EQ VAS score in the Untreated Cohort and by prescribed treatment. ^a^Because of the small sample size, data should be interpreted with caution. EQ VAS, EuroQol Visual Analogue Scale; LTP, long-term prophylaxis; OD, on-demand treatment; SD, standard deviation.

In the Untreated Cohort, the mean (SD) overall health status score “Today” was nearly 11 points higher than that “During the last untreated attack” (84.1 [11.0] and 73.0 [14.9], respectively) (Fig. 6). In the OD-only subgroup, the mean (SD) EQ VAS scores “Today” and “During the last untreated attack” were 86.7 (8.2) and 74.6 (9.8), respectively; in the LTP + OD subgroup, these values were 81.9 (12.9) and 71.7 (18.5), respectively.

#### HAEA-QoLv2 outcomes

**Fig. 7:**
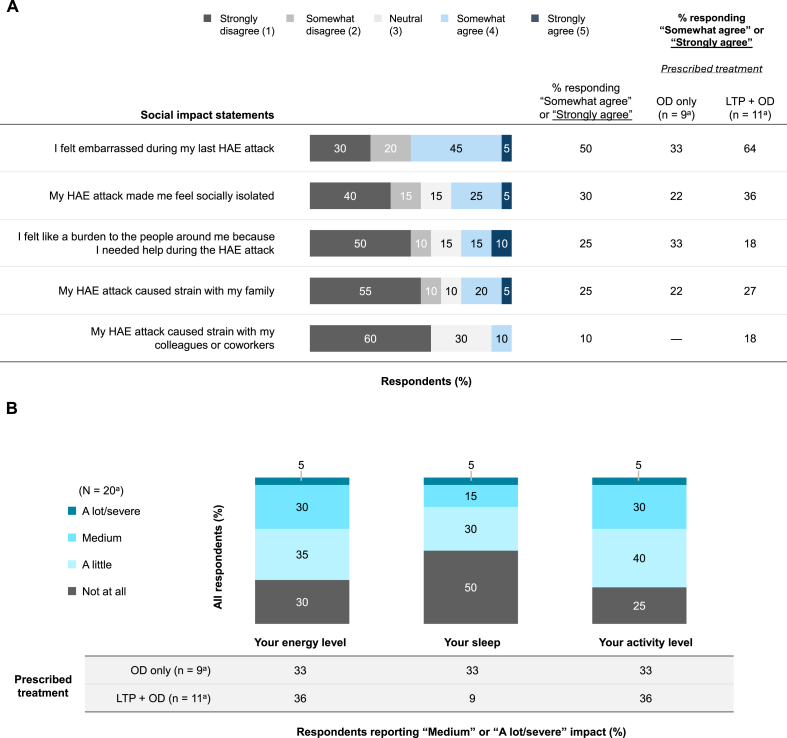
HAEA-QoLv2 outcomes for social impact (A) and physical impact (B) in the Untreated cohort. ^a^Because of the small sample size, data should be interpreted with caution. HAE, hereditary angioedema; HAEA-QoLv2, Hereditary Angioedema Association-Quality of Life Questionnaire version 2; LTP, long-term prophylaxis; OD, on-demand treatment.

Half (10/20) of respondents in the Untreated Cohort “Somewhat agreed” or “Strongly agreed” with the statement “I felt embarrassed during my last HAE attack” (Fig. 7A). One-third or fewer “Somewhat agreed” or “Strongly agreed” with each of the other social impact statements. In terms of physical impact, 35% (7/20), 20% (4/20), and 35% (7/20) of respondents reported that their last untreated attack had a “Medium” or “A lot/severe” negative impact on their energy level, sleep, and activity level, respectively (Fig. 7B).

A substantial proportion of patients in the LTP + OD subgroup (64% [7/11]) “Somewhat agreed” or “Strongly agreed” with the statement “I felt embarrassed during my last HAE attack” (Fig. 7A). A total of 33% (3/9) of patients in the OD-only subgroup and 36% (4/11) of those in the LTP + OD subgroup felt that their last untreated attack had a “Medium” or “A lot/severe” negative impact on their energy level, sleep, or activity level (Fig. 7B).

#### Adapted WPAI-GH

**Fig. 8:**
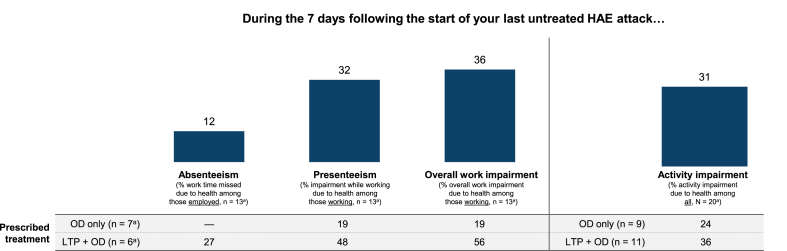
Adapted WPAI-GH in the Untreated Cohort. ^a^Because of the small sample size, data should be interpreted with caution. HAE, hereditary angioedema; LTP, long-term prophylaxis; OD, on-demand treatment; WPAI-GH, Work Productivity and Activity Impairment-General Health.

In the Untreated Cohort, the mean value for attack-related activity impairment was 31% (Fig. 8). Among the 13 respondents in the Untreated Cohort who were employed, the mean level of absenteeism, presenteeism, and overall work impairment due to the last untreated attack was 12%, 32%, and 36%, respectively. None of the 7 employed respondents in the OD-only subgroup reported absenteeism due to their last attack, but the mean level of presenteeism and overall work impairment were both 19%. Among the 6 employed respondents in the LTP + OD subgroup, the mean values for absenteeism, presenteeism, and overall work impairment were 27%, 48%, and 56%, respectively.

## Discussion

This US-based patient survey demonstrated that individuals with HAE experienced substantially/meaningfully lower HRQoL (as measured by adapted versions of the EQ-5D-5L, EQ VAS, and HAEA-QoL) and work productivity and increased activity impairment during and in the 7 days following their attacks, irrespective of whether those attacks were treated with OD. The negative impact of attacks on the HRQoL of patients with HAE is consistent with surveys performed in other countries.^6^^,^^8^, ^9^, ^10^, ^11^^,^^21^ In the present analysis, only 19% of patients treated their attack <1 h of onset,^19^ and as the time to OD increased, HRQoL generally worsened. As the time to OD increased, the proportion of patients with mild attacks also decreased,^19^ and as attack severity increased, HRQoL and work productivity during treated attacks decreased and activity impairment increased. Together, these data suggest that early treatment (ie, upon attack recognition) may mitigate the negative impact of attacks on patient well-being and functioning by reducing attack severity.

In the Treated Cohort, mean EQ-5D-5L index scores were substantially lower “During the last treated attack” than “Today” in both the OD-only (0.556 and 0.847, respectively) and LTP + OD (0.578 and 0.851, respectively) subgroups. Mean EQ VAS scores were also lower “During the last treated attack” relative to “Today” (OD-only subgroup: 58.5 and 79.0, respectively; LTP + OD subgroup: 54.6 and 73.2, respectively). In the Untreated Cohort, both EQ-5D-5L and EQ VAS scores were considerably lower “During the last untreated attack” than “Today” in the OD-only subgroup (mean EQ-5D-5L: 0.721 and 0.937, respectively; mean EQ VAS: 74.6 and 86.7, respectively) and the LTP + OD subgroup (mean EQ-5D-5L: 0.612 and 0.796, respectively; mean EQ VAS: 71.7 and 81.9, respectively). The mean values for overall work productivity and activity impairment in the LTP + OD subgroup of the Treated Cohort were 43% and 42%, respectively; the corresponding values in the LTP + OD subgroup of the Untreated Cohort were 56% and 36%. In comparison, in an analysis of the open-label extension to the phase 3 HELP trial of lanadelumab, these values were 12.1% and 18.5%, respectively.^29^ Collectively, these results underscore (1) how LTP users still experience substantial burden, not only in regards to attack severity, but also in terms of HRQoL, work productivity, and activity impairment during attacks and (2) how even patients in our Untreated Cohort (overall and in the OD-only and LTP + OD subgroups) had measurable reductions in HRQoL and work productivity and increases in activity impairment despite most (70%) attacks being perceived as mild. These findings further demonstrate the need to educate on the importance of treating all attacks and addressing barriers to early treatment.

Our survey revealed that the last treated attack negatively impacted the HRQoL of adolescents, with lower EQ-5D-5L and EQ VAS scores relative to adults. We also found that adolescents experienced substantially lower social and physical HRQoL during treated attacks (per the adapted version of the HAE-QoLv2). Despite the limited number of adolescents surveyed (n = 14), these observations are consistent with HRQoL research published on adolescents with other chronic conditions, such as asthma and type 1 diabetes.^30^^,^^31^ Notably, 29% of adolescents (vs 5% adults) visited the emergency department/hospital for OD; adolescents were also more likely to wait longer after attack onset to treat than adults (mean: 7.7 vs 3.2 h). This may be partly due to the fact only IV ODs are approved for adolescents in the United States. There is a need for an OD that is easier to administer and carry for adolescents, as this may enable the earlier treatment of attacks.

In terms of administration route, reductions in HRQoL and work productivity and increases in activity impairment were generally more pronounced among IV OD users than among SC OD users (mean EQ-5D-5L index score “During the last treated attack”: 0.480 vs 0.615). Consistent with this, IV OD users took longer to administer treatment following attack onset than SC OD users (mean: 5.8 vs 2.8 h). This suggests that an OD with an easier and more convenient route of administration may facilitate the early treatment of attacks.

One limitation of this analysis was the relatively small size of the Treated (N = 94) and Untreated Cohorts (N = 20), particularly when respondents were stratified into subgroups, limiting data interpretation and increasing the likelihood of sampling error. The sample size of this study is in keeping with recently published surveys analyzing HRQoL in patients with HAE.^32^, ^33^, ^34^, ^35^ Additionally, we did not adjust for any imbalances between subgroups or for confounders (eg, salaried vs hourly employment, income). Of note, surveys relying on self-reporting may be subject to recall bias. However, 88% of respondents across the Treated and Untreated Cohorts had their last attack in the prior 30 days, with >50% having their last attack within 2 weeks of the survey. In an effort to understand HRQoL during attacks, and similar to the analysis by Nordenfelt et al.,^20^ we adapted the recall period for the EQ-5D-5L to “During the last treated/untreated attack.” Like Nordenfelt et al., we found that as attack severity increased, mean EQ-5D-5L index scores decreased (Fig. 1, Fig. 5), indicating that the instrument remained sensitive to differences in attack severity despite the modification to the recall period. The adapted versions of the HAEA-QoLv2 (Figs. 3 and 7) and WPAI-GH (Figs. 4 and 8) were similarly sensitive.

## Conclusion

In this survey of US patients with HAE, acute attacks substantially affected health status, overall health, social and physical HRQoL, work productivity, and activity impairment, with a notable association between delays in administering OD and poorer outcomes. Declines in HRQoL and work productivity and increases in activity impairment were also seen during untreated attacks, despite most of these attacks being perceived by patients as mild. HRQoL and work productivity were substantially lower and activity impairment was substantially higher “During the last treated/untreated attack” relative to “Today” in both the OD-only and LTP + OD subgroups, emphasizing the significant burden of HAE attacks regardless of what treatment(s) are prescribed. We found that most patients delayed treating attacks, with IV OD users taking more time to administer treatment than SC OD users—a likely reflection of the ease of use of the respective agents. The negative impact of attacks on HRQoL, work productivity, and activity impairment may be reduced by increasing compliance with HAE guidelines, which recommend treating attacks as early as possible (ie, upon recognition), and by addressing barriers to OD in general and early treatment in particular.

## Abbreviations

C1INH, C1 esterase inhibitor; EQ-5D-5L, EuroQol–Five Dimensions–Five Levels; EQ VAS, EuroQol Visual Analog Scale; FDA, Food and Drug Administration; HAE, hereditary angioedema; HAEA, Hereditary Angioedema Association; HAEA-QoLv2, Hereditary Angioedema Association—Quality of Life Questionnaire version 2; HRQoL, health-related quality of life; IV, intravenous; LTP, long-term prophylaxis; OD, on-demand treatment; PRO, patient-reported outcome; SC, subcutaneous; SD, standard deviation; US, United States; WPAI-GH, Work Productivity and Activity Impairment–General Health.

## Author contributions

MO, PB, TJC, CR, HJW, PKA, VD, SC contributed to conception and design of the study. SD and JU contributed to the collection and assembly of data. MO, SD, JU, VD contributed the data analysis. MO, PB, TJC, CR, HJW, VD, TA, PKA, SC contributed to the interpretation of the results. All authors contributed to the review and editing of the manuscript. All authors provided final approval of the manuscript and are accountable for all aspects of the work.

## Ethics statement

The study was approved by the Advarra institutional review board. Respondents provided consent for their data to be used anonymously or in aggregate and were compensated for their time.

## Authors’ consent for publication

The authors gave their consent for publication.

## Funding

Funding for Outcomes Insights was provided by KalVista Pharmaceuticals (Cambridge, MA, USA).

## Declaration of competing interest

Dr. M. O'Connor is a speaker/consultant/advisor or researcher for KalVista Pharmaceuticals, Pharming, CSL, GSK, Blueprint, TEVA, AZ, Sanofi, Grifols, and Abbvie; and Chief Medical Officer of the CIIC.

Dr. P. Busse received consulting fees from Takeda, KalVista Pharmaceuticals, CVS Specialty, BioCryst, CSL, Behring, ADARx, Astria, and Pharvaris.

Dr. T.J. Craig received research support and is a consultant for CSL Behring, Ionis, Takeda, BioCryst, BioMarin, KalVista Pharmaceuticals, Pharvaris, Intellia, and Astria; received speaker fees from CSL Behring and Takeda; and travel support from CSL Behring, Takeda, and BioCryst.

Dr. C. Radojicic is a member of advisory boards for KalVista Pharmaceuticals, CSL, and Pharvaris.

Dr. J. Wedner has no conflicts to disclose.

Ms. S. Danese received consulting fees from KalVista Pharmaceuticals.

Ms. J. Ulloa received consulting fees from KalVista Pharmaceuticals.

Dr. V. Desai is a former employee of KalVista Pharmaceuticals.

Dr. T. Andriotti is a former employee of KalVista Pharmaceuticals.

Dr. P. Audhya is an employee of KalVista Pharmaceuticals.

Dr. S. Christiansen is a member of the US HAEA Medical Advisory Board, and participated in advisory boards for KalVista Pharmaceuticals, BioCryst, and CSL.
