# Supplementary material for: Impact of hereditary angioedema attacks on health-related quality of life and work productivity
Source: World Allergy Organ J. 2025 Jul 28;18(8):101083. doi: 10.1016/j.waojou.2025.101083 (PMC12320545; doi:10.1016/j.waojou.2025.101083)

Supplemental Table S1. Demographic and disease characteristics in the Treated Cohort and in the subgroup of employed respondents

	**Total (N = 94)**	**Employed (n = 42)**
**Current mean age, years (SD)**	39.4 (17.4)	39.0 (12.5)
**Mean age at diagnosis, years (SD)**	18.0 (12.6)	20.2 (13.0)
**HAE Type, %**		
Type 1	81	86
Type 2	19	14
**Gender, %**		
Female	7	73
**Race/ethnicity, %**		
White	87	83
Hispanic or Latino	9	10
Black/African American	3	2
American Indian/Alaskan Native	2	5
Asian	3	2
Other	1	–
**Payer coverage, %**		
Private or commercial	69	86
Medicare	16	5
Medicaid	14	2
Other	8	10
**Prescribed OD, n (%)**		
OD only	43 (46)	24 (57)
LTP + OD	51 (54)	18 (43)

HAE, hereditary angioedema; LTP, long-term prophylaxis; OD, on-demand treatment; SD, standard deviation.

Supplemental Fig. S1

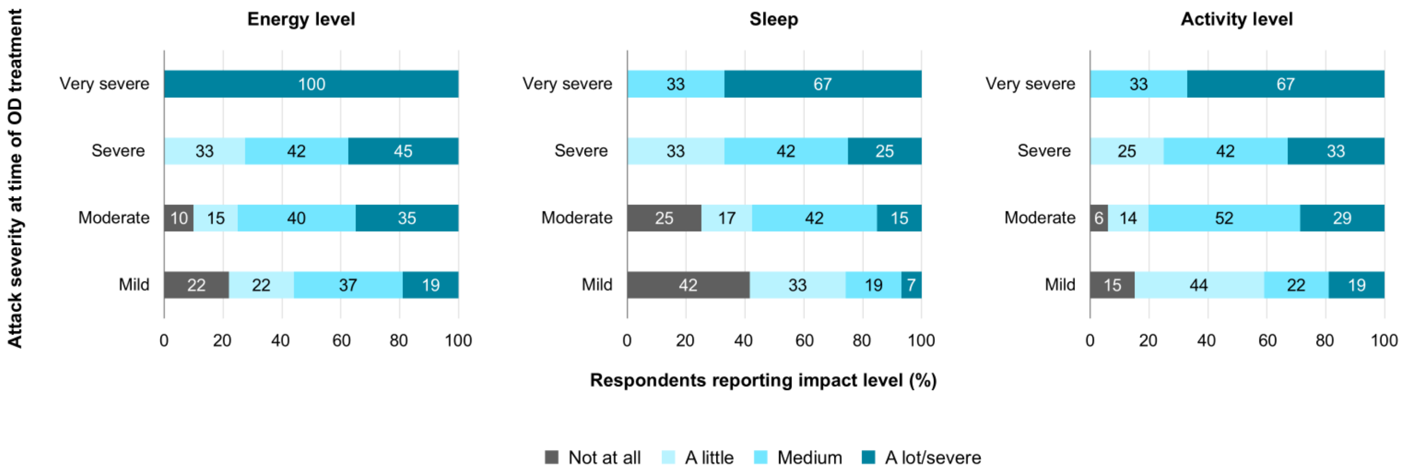

Supplement: Multimedia component 1 [file mmc1.pdf]
